# Molecular Remission Using Low-Dose Immunotherapy with Minimal Toxicities for Poor Prognosis IGHV—Unmutated Chronic Lymphocytic Leukemia

**DOI:** 10.3390/cells10010010

**Published:** 2020-12-22

**Authors:** Dipnarine Maharaj, Gayathri Srinivasan, Maria M. Abreu, Meng-Wei Ko, Anahid Jewett, Jacqueline Gouvea

**Affiliations:** 1 The Maharaj Institute of Immune Regenerative Medicine, Boynton Beach, FL 33437, USA; gayathrisrini27@gmail.com (G.S.); jgouvea@bmscti.org (J.G.); 2 Institute for NeuroImmune Medicine, Nova Southeastern University, Ft. Lauderdale, FL 33314, USA; mabreu1@nova.edu; 3 Division of Oral Biology and Medicine, The Jane and Jerry Weintraub Center for Reconstructive Biotechnology, UCLA, Los Angeles, CA 90095, USA; mengwei@g.ucla.edu (M.-W.K.); ajewett@dentistry.ucla.edu (A.J.)

**Keywords:** chronic lymphocytic leukemia, cancer treatment, immunotherapy, interleukin-2, natural killer cells

## Abstract

Chronic lymphocytic leukemia (CLL) accounts for 10% of hematologic malignancies. CLL is a malignancy of CD5+ B cells and it is characterized by the accumulation of small, mature-appearing neoplastic lymphocytes in the blood, bone marrow, and secondary lymphoid tissues. In the present case, a middle-aged female patient with poor prognosis unmutated IGHV CLL achieved cytogenetic and molecular remission with minimal adverse events following six cycles of low dose recombinant human IL-2 (rIL-2) in combination with low dose targeted venetoclax. Personalized low dose rIL-2 in combination with either lenalidomide or venetoclax mediates natural killer stimulation and is an effective non-toxic immunotherapy administered in the outpatient setting for poor prognosis CLL.

## 1. Introduction

Chronic lymphocytic leukemia (CLL) is a neoplasm of small mature B cells and is the most common leukemia diagnosed in adults. CLL comprises two major subsets with unmutated (U-CLL) or mutated (M-CLL) immunoglobulin heavy chain variable region genes (IGHV) with an origin from the pre-germinal center (GC) or post-GC CD5+ B cells, respectively [1,2]. The classification of either the U-CLL or M-CLL status has clinically different outcomes with the mutated status having a better prognosis and mean overall survival of 293 months while U-CLL is associated with a poorer prognosis and has a shorter survival (mean OS = 95 months) [3].

CLL is diagnosed by detecting clonal CD5+ B lymphocytes at a count of ≥5000 μL in the peripheral blood. At the time of CLL diagnosis, almost all patients have quantitative and qualitative defects within the innate and adaptive immune response system [4,5]. Innate immune cells including neutrophils, natural killer (NK) cells, and monocytes have functional defects. The circulating monocytes increase by >60% in patients with CLL, but such cells are associated with immunosuppressive properties [6,7]. Although refractory to stimuli from normal B cells, monocytes express an immunosuppressive M2 macrophage-like phenotype in response to CLL-derived soluble stimuli [6,8,9]. Absolute CD4+ and CD8+ T cell counts increase in early-stage disease, but these numbers decrease with disease progression as does the function, and in particular, there is markedly impaired helper activity [10]. The expansion of regulatory T (CD4+CD25+) cells in late-stage CLL contributes to immune suppression. B CLL lymphocytes directly suppress the function of normal T cells and B cells by cell release of tumor-derived soluble factors [11]. CLL cells also have functional properties of B regulatory cells, which negatively regulate adaptive and innate immune responses through soluble factors, predominantly IL-10 [9,12].

Current therapy for CLL combines chemotherapy agents, such as fludarabine, cyclophosphamide, bendamustine with rituximab, and other anti-CD20 monoclonal antibodies. Although they have proven efficacy in symptomatic control of disease, these chemo- immunotherapies have a negative impact on immune function. As such, the consequences of these treatments in a patient with CLL is a net accumulation of infectious episodes over time and a failure to reverse the underlying features of immune suppression. Response rates in CLL for these treatments initially seem to show positive results. However, molecular remission in CLL is often very rare. With evolving novel targeted CLL therapies, ibrutinib (Bruton’s tyrosine kinase (BTK) inhibitor) demonstrated improved clinical outcomes as well as superior overall and progression-free survival relative to fludarabine, cyclophosphamide, and rituximab (FCR) among younger patients [13,14]. Ibrutinib also targets interleukin-2-inducible kinase within T cells, suggesting possible off-target effects that could contribute to the disruption of immune homeostasis in the longer term [15]. Emerging targeted medications, including venetoclax and idelalisib have improved the therapeutic landscape of CLL [16]. Randomized controlled studies performed with idelalisib and rituximab have shown significant improvement in progression free and overall survival rates in comparison to placebo [17]. Phase 2 studies assessing the efficacy of venetoclax monotherapy in relapsed or refractory CLL have shown 57% of patients achieving partial remission (PR) while 2% achieved complete remission (CR) [16]. Additionally, studies evaluating minimal residual disease (MRD) status in CLL patients with 17p deletions receiving standard dose venetoclax monotherapy for a median of 23.1 months have shown that 30% of patients achieved undetectable minimal residual disease (u-MRD defined as less than 1 CLL cell per 10,000 lymphocytes [18]) in peripheral blood [16].

Despite promising results, current therapies are not without severe adverse events. FCR carries the risk of hemolytic anemia and secondary malignancies, while ibrutinib can cause frequent fatal events, including bleeding, cardiac arrhythmias, neutropenia, and infections [14]. Idelalisib has been associated with severe adverse reactions such as diarrhea, colitis, pneumonia, and sepsis [17]. The most frequent serious adverse events with standard dose venetoclax are pneumonia, sepsis, and severe life-threatening neutropenia observed in 40% of patients [16]. Immunotherapies such as CAR-T cells use the patient’s immune system to induce therapeutic responses. However, responses in CLL were often disappointing due to the profound immune dysregulation by CLL cells [19].

Natural killer (NK) cells are highly cytotoxic lymphocytes of the innate immune system that are capable of controlling and limiting the spread of tumors and infections and have shown promising results in anti-tumor immunotherapy [20]. NK cells are phenotypically defined by the expression of CD56 and the lack of CD3-TCR complex [21]. Within this population, NK cells are further divided into subsets based on CD16 and CD56 expression levels [21,22]. CD56 (high) CD16 (-) cells are specialized for cytokine production and have low levels of perforin. CD56 (high) cells have a strong affinity for IL-2 and proliferate efficiently in response to its subcutaneous administration [22]. CD56 (high) cells may regulate the function of other cells and are known to induce differentiation of tumor cells [23]. The CD56 (low) CD16 (high) NK cell subset mediates natural and antibody-dependent cellular cytotoxicity, exhibiting high levels of perforin, and enhanced killing. In patients with CLL, overall NK cell numbers are increased, but their function is severely depleted [20].

IL-2, a cytokine with pleiotropic effects, is essential for the proliferation and activation of many cell types, including NK cells [24]. When activated by IL-2, NK cells acquire an enhanced killing function and can recognize a broader range of targets and kill with an increased lytic potential [24]. Therapeutic uses of IL-2 have utilized two different strategies by varying the dose of IL-2; ultralow doses to reduce autoimmune responses through regulatory T cell stimulation and low doses to augment NK cell-mediated antitumor immune responses [25,26]. This dualistic anti and pro-immune function of IL-2 based on dosing and frequency can be utilized in a personalized therapeutic approach in immune-oncology in which NK cell antitumor responses can be augmented.

The novel combination approach of low dose immunotherapy combined with low dose targeted therapy can amplify anti-tumor NK and immune functional activity while directly destroying cancer cells and minimizing toxicity to the patient [26]. Lenalidomide shows anti-tumor immunity against CLL by repairing T cell defects, downregulating T cell inhibitory molecules in CLL [27] and enhancing T cell motility [28]. Lenalidomide also induces NK cell expansion and activation [29]. Venetoclax promotes the killing of CLL cells through the inhibition of BCL-2, which in the pathogenic state enhances the survival of leukemic cells [30].

In this case report, we describe a middle-aged female patient with U-CLL following treatment with ibrutinib, which had to be discontinued due to severe adverse reactions. The patient achieved cytogenetic and molecular remission with minimal adverse events following multiple cycles of low doses of rIL-2 in combination with low dose targeted therapy.

## 2. Results

### Case Report

**Figure 1:**
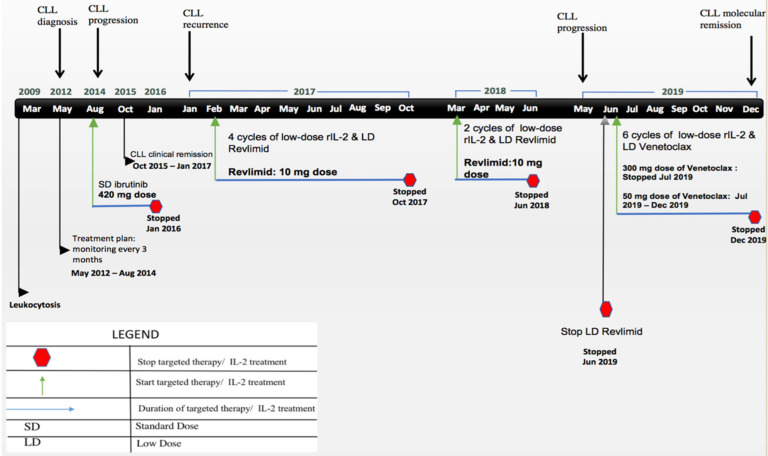
Key timeline information. The black arrow indicates time of CLL diagnosis and progress. Green arrow indicates treatment initiation. Patient went on and off treatments. In August 2014, ibrutinib treatment was started at 420 mg once daily. In February 2017, immunotherapy consisted of daily low-dose subcutaneous rIL-2 injections of 10–20,000 IU/kg for 5 days per week in addition to oral low dose lenalidomide 10 mg per day. In March 2018, two cycles of low-dose rIL-2. In June 2019, immunotherapy consisted of low-dose subcutaneous rIL-2 injections and low-dose venetoclax 50 mg per day. Blue arrows indicate the length of treatment. Red octagons indicate when the patient would stop treatment.

A 56-year-old woman first attended our clinic in October 2015 with a history of B- CLL. (timeline of the key information is summarized in Figure 1). In March 2009, she had a facelift procedure, and blood counts revealed a leukocytosis post-operatively. In May 2012, the patient developed enlarged neck lymph nodes. Blood counts revealed a white cell count of 19 K/μL with 8.31 K/μL abnormal lymphocytes measured by flow cytometry with a monoclonal lambda B cell population co-expressing CD5 and CD23 consistent with CLL. Other positive markers included CD19, CD20, CD11c, and CD45. Fluorescence in-situ hybridization (FISH) analysis in July 2012 showed a heterozygous deletion of the D13S319 locus (13q14.3) in 33.7% of cells (normal < 3.1%). CT scans showed extensive pathologic anterior and posterior cervical adenopathy. The treatment plan was observation and monitoring with blood counts and clinical examination every three months. In August 2014, the patient developed severe fatigue, dizziness, and anemia, requiring a transfusion of two units of packed red blood cells. The patient was started on treatment with Imbruvica (ibrutinib) 420 mg once daily.

In October 2015, the patient attended our clinic for evaluation of her disease. She was in clinical remission. Routine blood work showed white blood count: 7.6 K/μL, hemoglobin: 14.5 g/dL, platelets: 143 K/μL. In January 2016, with the onset of adverse reactions of severe skin rash on her arms and scalp tenderness, ibrutinib was discontinued.

In late December of 2016 through early January of 2017, the patient had a recurrence of enlarged neck lymph nodes. Blood counts revealed an elevated WBC of 27.1 K/μL and a low platelet count of 121 K/μL with flow cytometry showing abnormal CLL cells at 9.27 K/μL. FISH showed a heterozygous deletion of the D13S319 locus (13q14.3). Molecular pathology showed unmutated IGHV, indicative of aggressive disease (Table 1).

**Figure 2:**
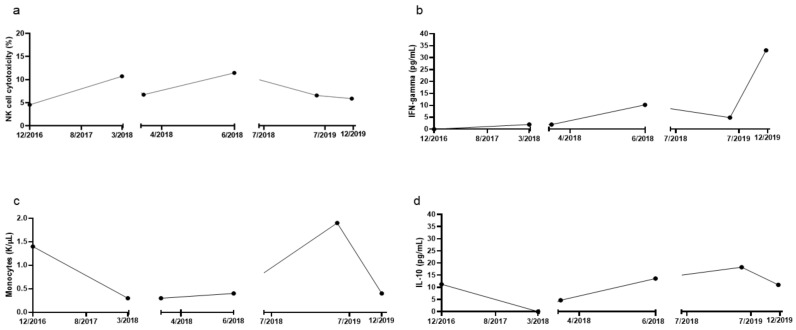
Changes in immune cell function and cytokine secretion in response to r-IL-2 immunotherapy. (**a**) Percent of NK cell cytotoxicity as measured in a whole blood chromium release assay. (**b**) IFN-gamma levels (ELISA) in peripheral blood. (**c**) Absolute monocyte cell count monitored by complete blood count. (**d**) IL-10 levels (ELISA) in peripheral blood.

Treatment options discussed included responses and side effects of fludarabine, cyclophosphamide, rituximab, bendamustine, CD20 monoclonal antibodies, targeted therapy including venetoclax and idelalisib, bone marrow transplantation, CAR-T cell therapy, no therapy, or experimental personalized low-dose immunotherapy. The patient refused chemotherapy or treatments with potentially severe side effects. She gave informed consent for experimental personalized low-dose immunotherapy, including publication of results. Peripheral blood immune evaluation showed an increase in absolute monocyte count of 1.355 K/μL (normal range: 0.55–1.00 K/μL). While NK cells were increased at 628 cells/μL (normal range: 98–294 cells/μL), there was decreased functional NK cell cytotoxicity to 4.56% (normal range: 16–39.9%) (Figure 2a). CD8+ T cells were 364 cells/μL (normal range: 219–731 cells/μL), CD4+ T cells levels were high at 1210 cells/μL (normal range: 599–1199 cells/μL), and CD4:CD8 ratio of 3.32. The median percentage of regulatory T cells (CD4+CD25+) was low at 0.18% (normal range: 4.93–7.29%), and NKT cells numbers at 1014.0 cells/μL (normal range: 593–1375 cells/μL). Plasma cytokines showed low levels of interferon-gamma (IFN-g) at 0 pg/mL and elevated levels of IL-10 11.25 pg/mL (Figure 2b,d).

In February 2017, the patient began low-dose immunotherapy to selectively improve the immune dysregulation of CLL. Cycles consisted of daily low-dose subcutaneous rIL-2 injections of 10–20,000 IU/kg for five days per week in addition to oral low-dose lenalidomide 10 mg per day. Variation of the duration, dosing and frequency of administration of cycles of rIL-2 was based on peripheral blood immune panel results. Disease progression was monitored through peripheral blood counts, flow cytometry, FISH, and molecular analysis.

By October 2017, the patient had received four cycles of rIL-2 injections, which she tolerated well with no toxicities. Flow cytometry showed significant improvements with a decrease in abnormal CLL cells to MRD of 0.11 K/μL and FISH was negative for the heterozygous deletion of the D13S319 locus (13q14.3). Molecular pathology showed a mutated IGHV. However, there was persistence of SF3B1 mutation, which is associated with a poor prognosis. Further treatment with low-dose rIL-2 was recommended but the patient did not comply.

In March of 2018, flow cytometry showed that the absolute number of abnormal CLL cells were 0.08 K/μL, FISH showed recurrence of heterozygous deletion of the D13S319 locus (13q14.3). Molecular pathology showed progression to an unmutated IGHV and persistence of the SF3B1 mutation. Peripheral blood immune evaluation showed that her NK cell function was 6.74% and this improved to 11.45%% in June of 2018 in response to two cycles of low-dose rIL-2 (Figure 2a). In addition, in June of 2018, molecular pathology showed persistence of unmutated IGHV and the SF3B1 mutation. Further low-dose rIL-2 was recommended, but the patient was feeling well and chose not to have treatment.

In May of 2019, the patient had a recurrence of fatigue and enlarged neck lymph nodes. Blood counts revealed an elevated WBC of 56.7 K/μL and low platelets of 123 K/μL. Flow cytometry showed a major increase of 49.6 K/μL abnormal CLL cells and FISH showed a heterozygous deletion of the D13S319 locus (13q14.3). Molecular pathology revealed a persistent SF3B1 mutation and unmutated IGHV. Immune panel results showed that her NK cell function (6.57%) had also decreased during the period of time when the patient was off treatment with low dose rIL-2 (Figure 2a).

In June of 2019, the patient had extreme fatigue caused by Coombs positive autoimmune hemolytic anemia (AIHA) secondary to CLL. The immune hemolysis resolved in response to prednisone and she significantly improved with hemoglobin levels rising from 5.2 g/dL during the peak of hemolysis, to 10.7 g/dL. As a precaution, further treatment with low dose lenalidomide was discontinued because of the risk of hemolysis reported with lenalidomide at standard doses. The patient agreed to standard dose monotherapy with venetoclax with weekly dosage increase (20 mg, 50 mg, 100 mg, 200 mg, and 300 mg). At the 300 mg dose of venetoclax the patient experienced side effects of severe neutropenia, (0.1 K/μL) requiring granulocyte-colony stimulating factor administration, as well as thrombocytopenia (100 K/μL). Venetoclax was discontinued. After her blood counts had improved, the patient requested restarting immunotherapy with low-dose rIL-2. A low-dose of venetoclax at 50 mg per day was added to minimize its toxicity.

In October 2019, the patient was asymptomatic. She had received four cycles of low-dose rIL-2 and daily venetoclax 50 mg with minimal toxicity. Blood counts revealed a WBC of 15.4 K/μL and normal platelets of 160 K/μL. Flow cytometry showed a major response to treatment with MRD of 0.33 K/μL absolute abnormal CLL cells and FISH showed a heterozygous deletion of the D13S319 locus (13q14.3). Molecular pathology showed mutated IGHV. However, there was a persistent SF3B1 and ATM mutation.

In December of 2019, after two further cycles of low dose rIL-2 and low dose venetoclax, the patient experienced minimal side effects. Blood counts revealed normal white blood count of 4.72 K/μL, hemoglobin of 13.60 g/dL with a low platelet count of 137 K/μL. Flow cytometry showed uMRD with no abnormal CLL cells. FISH showed no evidence of previously observed deletions. Molecular pathology was negative for previously seen mutations. Peripheral blood immune evaluation showed an absolute monocyte count of 0.36 K/μL, NK cells normal at 139 cells/μL but NK cell cytotoxicity reduced at 5.89% (Figure 2a). However, the patients NK cell subsets (CD56high, CD16-) showed increased levels at 14.21%, (CD56high, CD16+) at 14.36%, (CD56low, CD16+) low at 4.31% and (CD56low, CD16-) increased at 12.86%. Other cell populations including CD4+ T cells were normal at 625 cells/μL, CD8+ T cells decreased to 129 cells/μL, resulting in a CD4:CD8 ratio of 4.84. Analysis of median percentage of regulatory T cells (CD4+CD25+) revealed low levels at 2.08% and decreased NKT cells numbers at 530 cells/μL. Plasma cytokines showed an increase in IFN-g at 33.09 pg/mL and a decrease in IL-10 at 11.02 pg/mL.

**Figure 3:**
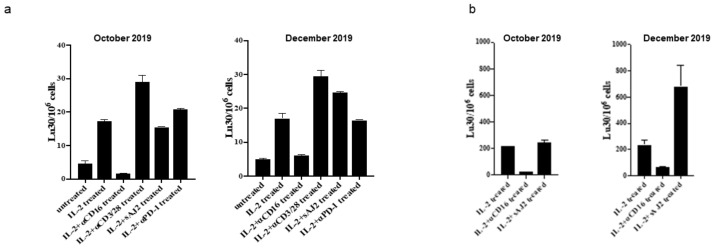
Cytotoxicity of NK cells improved substantially from October to December. PBMC (**a**) and NK cells sorted from PBMCs (**b**) were left untreated or treated with IL-2 (1000 U/mL), the combination of IL-2 and anti-CD16 mAb (3 µg/mL), IL-2 and anti-CD3/28 antibody (25 mL/mL), IL-2 and sonicated probiotics (sAJ2) (bacteria: PBMC, 20:1) or anti-PD-1 antibody (10 ng/mL) for 18 h. NK cell mediated cytotoxicity using a standard 4-h chromium release assay against oral squamous cell carcinoma stem cells (OSCSCs) were performed and the lytic unit 30/106 cells were measured.

Further in vitro functional analyses as described [31] were performed on the patient’s peripheral blood in October 2019 and compared to similar analyses performed in December 2019. The percentages of all different subsets of immune effectors improved and resembled that of the healthy controls. From October 2019 to December 2019,the largest increase detected was in CD3+ T cells, which went from 17% to 51%. B cells (CD19+) significantly improved from 70.2% to within the normal range of 13.76%. In a chromium release assay, cytotoxicity of NK cells within PBMCs improved (Figure 3a). In NK cells negatively selected from the peripheral blood and cytotoxicity measured against oral squamous cell carcinoma stem cells (OSCSCs), the levels of cytotoxicity improved substantially in all treated samples and of particular interest the levels of NK activity when treated with AJ2 bacteria showed a substantial 2.8 fold increase (Figure 3b).

**Figure 4:**
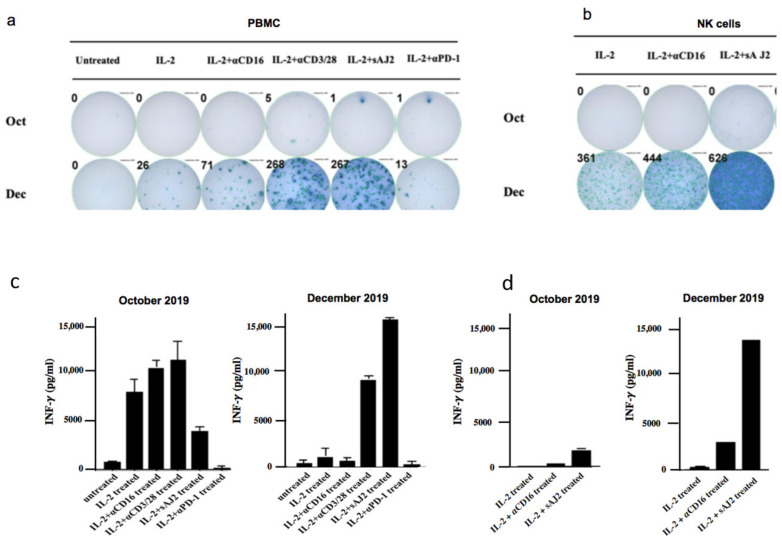
Significant improvement in IFN-g spots and secretion from both PBMCs and NK cells. PBMC and NK cells sorted from PBMCs were left untreated or treated with IL-2 (1000 U/mL), the combination of IL-2 and anti-CD16 mAb (3µg/mL), IL-2 and anti-CD3/28 antibody, IL-2 and sonicated probiotics (sAJ2) (bacteria: PBMC, 20:1) or anti-PD-1 antibody (10 ng/mL) for 18 h. ELISPOT (**a**,**b**) was conducted to determine the numbers of cells demonstrating the IFN-g spots represented by the number and size of IFN-g spots. Supernatants were also collected after 18 h of the treatment and ELISA (**c**,**d**) was performed to measure the concentration of IFN-g secreted within the cultures.

Significant improvement in the IFN-g spots and IFN-g release from both PBMCs and NK cells were seen (Figure 4a,b). When the levels of IFN-g spots were determined by ELISPOT assay within PBMCs and the NK cells, there were significant increases in the number of detected spots from October 2019 to December 2019 in all treated samples. (Figure 4a,b). When the levels of IFN-g secretion were determined by ELISA within sorted populations of NK cells, there were significant increases in the amount of IFN-g secretion from October 2019 to December 2019 in all treated samples. (Figure 4d).

**Figure 5:**
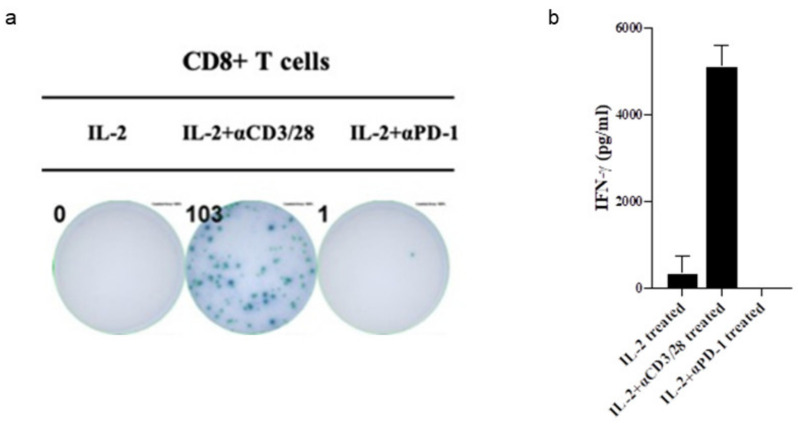
Significant increase of IFN-g secretion by anti-CD3/28 antibody treated CD8+ T cells. CD8+ T cells were sorted from PBMCs and treated with IL-2, the combination of IL-2 and anti-CD3/28 antibody (25µL/mL) or anti-PD-1 antibody (10 ng/mL) for 18 h before using in the ELISPOT (**a**) to determine the number of IFN-g secreting cells. Culture supernatants were collected for ELISA assays (**b**) to measure the concentrations of IFN-g secretion from treated CD8+ T cells.

When the levels of IFN-g secretion were determined by ELISPOT and ELISA (Figure 5) within sorted populations of CD8+ T cells, significant increases in the amounts of IFN-g secretion as well as IFN-g spots from anti-CD3+/anti-CD28 treated CD8+ T cells were observed. These results suggest significant improvements in the quantitative and qualitative defects within the innate and adaptive immune response system when the patient was treated with multiple sequential cycles of low dose rIL-2 and low dose venetoclax.

Nine months after completion of the rIL-2 treatment and low-dose targeted therapy, repeat evaluation and testing showed that the patient maintained complete clinical, hematological, cytogenetic and molecular remission. Flow cytometry showed no abnormal CLL cells. FISH analysis was normal with no detection of previously observed deletions. Molecular pathology showed no abnormal mutations. Current testing shows that the patient remains in remission.

## 3. Discussion

We report a case of cytogenetic and molecular remission with personalized low-dose immunotherapy treatment using rIL-2 and low-dose targeted therapy to increase NK cell function in a patient with poor prognosis U-CLL. The patient experienced minimal side effects throughout her treatment with low dose rIL-2 and low dose targeted therapy. At the time of initiation of low dose rIL-2, the patient had received the standard of care with ibrutinib, which had to be discontinued because of intolerable toxicity.

Based on experimental data and treatment results in malignancies [26,32], we personalized the dose and schedule of low-dose rIL-2 by monitoring the immune effector cell levels and activity in the peripheral blood before and after treatment cycles. The treatment plan was that the patient would receive continuous cycles of rIL-2 followed by a small gap after each treatment cycle to minimize IL-2 related side effects while increasing the activity of immune effector cells. However, the patient interrupted this treatment plan several times. After the initial four cycles, she achieved a good response. Then there was a delay followed by a short period of two cycles and a further delay of over a year, resulting in disease progression. Subsequently, when the patient finally completed the planned protocol, she achieved molecular remission after six uninterrupted cycles.

The patient tolerated low dose lenalidomide without any apparent toxicity. Nevertheless, since at standard doses of lenalidomide a reported side effect is warm AIHA [33], the low dose lenalidomide was discontinued after the patient developed AIHA which is a well-established complication of CLL. The patient was then treated with standard-dose venetoclax monotherapy but after the development of severe toxicity, she then chose to continue treatment with the personalized dose and schedule of low-dose IL-2 and low dose venetoclax.

The dualistic approach of low dose immunotherapy with the addition of low dose targeted therapy can amplify anti-tumor NK cell functional activity while directly destroying cancer cells. Standard dose venetoclax has been shown to reduce immunosuppressive regulatory T cells in the lymph node environment while sparing nonmalignant lymphocytes and promoting immune recovery through the restoration of NK cell function [34]. In patients with CLL, venetoclax acts a BCL2 inhibitor to promote rapid killing of CLL cells [30]. At the 400 mg dose, severe side effects such as neutropenia and thrombocytopenia occur, however doses as low as 20 and 50 mg are strong enough to demonstrate induction of apoptosis in some patients with minimal adverse events [30,35]. Our patient experienced severe adverse effects at a standard dose (300 mg) of venetoclax, but tolerated low dose (50 mg) well with no side effects. While there is no evidence to indicate that CLL patients achieve molecular remission with low dose Venetoclax monotherapy, in our patient the novel combination of the personalized dose and schedule of low-dose IL-2 and low dose venetoclax resulted in molecular remission. Evaluation of her peripheral blood showed restoration of the functional activity of her NK cells. The relative contributions to the improvement in NK cell function of venetoclax or lenalidomide in combination with IL-2 are not known since these were not evaluated in our in-vitro testing.

Before starting any IL-2 and targeted therapy treatments, the patient had an abnormal NK cell function of 4.56%, which then increased in response to treatment and abnormal CLL cells decreased. A significant reduction in NK cell function were due to the gaps in treatment, which corresponded to increases in abnormal CLL cells as depicted in Figure 2c. When the patient was in complete molecular remission, there was an overall increase in NK cells and subsets. The CD56 (high) subset had greatly increased to 14.21%. One of the cytokines produced by CD56 (high) NK cells is IFN-g, which has immune regulatory activity and anti-viral activity [36]. In response to treatment, the patient’s plasma IFN-g levels increased from undetectable levels to 33.09 pg/mL as shown in Figure 2b. This elevated response coincided with the increases in NK cells and subsets. Further in vitro functional analyses of the patient’s peripheral blood in October 2019 and in December 2019 showed that the most substantial increases were found in IFN-g production in which both the levels of PBMC, NK and CD8+ T cells had increased numbers expressing IFN-g as well as increase in secretion of IFN-g. This finding is very important since it indicates that augmented increase in IFN-g secretion may be an important driver of differentiation of the tumors and curtail tumor growth [23].

Flow cytometry, FISH, and molecular pathology were taken periodically throughout the patient’s treatment to monitor disease progression. In January of 2017, prior to starting low-dose rIL-2 the patient expressed 9.27 K/μL abnormal CLL cells, a heterozygous D13S319 locus (13q143) in 71% of cells and an unmutated IGHV. Recent studies showed that a mutated IGHV mutation corresponded with 9.2–18.9 years of progression-free survival (PFS) in comparison to an unmutated IGHV, which led to 1–5 years of PFS [37]. Overall survival (OS) is observed from 17.9 to 25.8 years in patients with a mutated state, compared to 3.2 to 10 years in the unmutated state [37]. After four continuous cycles of the dualistic low dose IL-2 and targeted therapy, the patient was able to achieve and remain in the mutated state until a gap in treatment. With the persistence of the combined treatment, FISH showed no chromosomal deletions and flow cytometry showed undetectable minimal residual disease of CLL. (uMRD) (Table 1). The significance of the MRD status in CLL has grown steadily throughout the years. Several randomized clinical trials (RCT) have shown that MRD is an independent predictor of OS and PFS [18]. Our patient was able to achieve uMRD after six uninterrupted cycles of rIL-2 and venetoclax and she was able to maintain that status after the end of treatment (Table 1).

In our patient the dualistic low dose IL-2 and targeted therapy was associated with a reduction in absolute monocyte counts and IL-10 levels which most likely contributed to the improvement in disease outcome. Elevated levels of circulating monocytes can contribute to CLL cell survival and proliferation by increasing myeloid derived suppressor cells (MDSC), allowing CLL cells to evade immune surveillance which is associated with a poor outcome [38]. Patients with CLL have higher levels of IL-10 than healthy individuals. IL-10, a T helper 2-type cytokine, has immunosuppressive properties as it inhibits T cell proliferation and macrophage cytokine production. In vitro studies have shown that the suppression of IL-10 can promote an anti-tumor immune response in CLL patients [39]. At baseline, the patient’s absolute monocyte and IL-10 levels were elevated at 1.355 K/μL and 11.25 pg/mL respectively, which corresponded with the patient’s elevated number of abnormal CLL cells. With continuous treatments of IL-2 and targeted therapy, both monocyte and IL-10 levels decreased as well as abnormal CLL cells. Large gaps in the patient’s treatment corresponded with increases in monocyte, IL-10 and abnormal CLL levels and showed reductions when the patient resumed treatment.

This report describes a previously unreported approach for treating U-CLL by using measurements of NK cell function and its subsets, IFN-g and IL-10 levels, absolute monocyte count, flow cytometry, FISH analysis, and molecular pathology while tailoring the dose and frequency of IL-2 and targeted therapy in response to the immune effector cells. Our report serves as a basis for a precision personalized low-toxicity immunotherapeutic option in patients with poor prognosis disease.

## 4. Conclusions

U-CLL is associated with a poor prognosis. Our data showed that personalized treatment with low-doses of rIL-2 and targeted therapy was associated with activation of effector immune and NK cells to abnormal CLL cells and resulted in molecular remission with minimal side effects. We were able to follow the progress and treatment by monitoring the peripheral blood CLL cells and NK cell immune activation and tailor the treatment dose and frequency to achieve the desired outcome for the patient.

## 5. Future Perspective

This case report shows that personalized immunotherapy with rIL-2, in combination with targeted therapy, resulted in a complete molecular and cytogenetic remission with minimal adverse events in a patient with poor prognosis U-CLL. This unique approach is an important contribution and supports the need for future research concerning these combined therapeutic options to improve outcomes and minimize side effects in patients with cancer.

## Figures and Tables

**Table 1:** Results of FISH, abnormal CLL cells, and IGHV mutation status changes in response to treatment.

Date of Visit	FISH 13q14.3 (Normal < 3.1%)	Absolute Number of Abnormal CLL Cells * K/μL	Detected Abnormalities
31 January 2017	71%	9.27 (59%)	ZAP-70 positive Unmutated IGHV: 0.3%
7 April 2017	52%	1.91 (49%)	ZAP-70 positive SF3B1 mutation: detected TP53 deletion: not detected NOTCH1: not detected Unmutated IGHV: 1.7%
8 June 2017	51%	0.27 (16%)	ZAP-70 positive SF3B1 mutation: detected TP53 mutation: not detected NOTCH1: not detected MYD88: not detected Unmutated IGHV: 1.0%
4 August 2017	26%	0.13 (9%)	SF3B1 mutation: detected TP53 mutation: not detected NOTCH1: not detected MYD88: not detected Mutated IGHV: 6.0%
18 October 2017	Not detected	0.11 (10%)	SF3B1 mutation: detected TP53 mutation: not detected NOTCH1: not detected MYD88: not detected Mutated IGHV: 9.5%
27 March 2018	24%	0.08 (5%)	SF3B1 mutation: detected TP53 mutation: not detected NOTCH1: not detected MYD88: not detected Unmutated IGHV: 0.34%
5 June 2018	21%	0.62 (27%)	Unmutated IGHV: 0%
17 May 2019	93%	49.6 (79%)	SF3B1 mutation: detected TP53 mutation: not detected NOTCH1: not detected MYD88: not detected Unmutated IGHV: 0.3%
26 August 2019	52%	0.33 (55%)	SF3B1 mutation: detected ATM mutation: detected TP53 mutation: not detected NOTCH1: not detected MYD88: not detected Unmutated IGHV: 0%
3 October 2019	7.5%	0.33 (9.6%)	SF3B1 mutation: detected ATM mutation: detected TP53 mutation: not detected NOTCH1: not detected MYD88: not detected Mutated IGHV: 12.8%
6 December 2019	Not detected	No abnormal CLL cells (0%)	No mutations
12 March 2020	Not detected	No abnormal CLL cells (0%)	No mutations
29 September 2020	Not detected	No abnormal CLL cells (0%)	No mutations

* Absolute number of CLL cells followed by percentage of total lymphocytes in parentheses.
